# Quality evaluation of selected expired fluoroquinolones medicines obtained from the public hospitals in Jimma zone, Oromia regional state, Ethiopia

**DOI:** 10.3389/fmed.2024.1420146

**Published:** 2024-08-07

**Authors:** Habtamu Getahun, Sileshi Belew, Gemmechu Hasen, Guta Tefera, Yesuneh Tefera Mekasha, Sultan Suleman

**Affiliations:** ^1^Pharmaceutical Quality Assurance and Regulatory Affairs, Oromia Regional Health Bureau, Addis Ababa, Oromia, Ethiopia; ^2^School of Pharmacy, Institute of Health Science, Jimma University, Jimma, Oromia, Ethiopia; ^3^School of Pharmacy, Pharmaceutical Quality Assurance and Regulatory Affairs, Wolkite University, Wolkite, Ethiopia; ^4^Pharmaceutical Science, Pharmaceutical Quality Assurance and Regulatory Affairs, University of Gondar, Gondar, Ethiopia

**Keywords:** expired medicines, ciprofloxacin, norfloxacin, assay, identity, dissolution pharmacopeia, failure mode, effect analysis

## Abstract

**Background:**

The problem of medicine expiration presents a notable obstacle, resulting in considerable financial losses. Nevertheless, there is currently limited data indicating that certain medications do not experience a significant decrease in effectiveness after their expiration date. Therefore, the aim of the study was to assess the physico-chemical quality of expired fluoroquinolone antibiotics.

**Methods:**

The expired samples of fluoroquinolone antibiotics were purposively collected from public hospitals in the Jimma zone of the Oromia regional state, Ethiopia. A World Health Organization quality evaluation sampling strategy was employed. Then, simple random sampling techniques were utilized for the selection of tablets for the laboratory quality control test. The assay, identification, and dissolution were performed in accordance with the United States Pharmacopeia (USP) guidelines, as well as failure mode and effect analysis (FMEA) techniques.

**Results:**

The finding revealed that about 100% (7/7) expired samples passed pharmacopeia quality specifications for identity and assay tests. However, of the seven expired brands, about 14.3% (1/7) of the sample (Code-002) was unable to release its API content within the USP criteria of 30 min. The risk-based quality evaluation revealed that assay was the most critical quality attributed to ciprofloxacin tablets (RPN = 189), followed by identity (RPN = 100). Assay was also the most critical quality attribute (RPN = 378), followed by identity (RPN = 100) for Norfloxacin tablets. The risk-based desirability function approach showed that 75% (3/4) of ciprofloxacin products were of good quality, and 25% (1) were found to be of acceptable quality, while the desirability function of norfloxacin tablets was found to be excellent 1 (33.3%), good 1 (33.3%), and acceptable 1 (33.3%).

**Conclusion:**

The study revealed that medications can maintain their quality beyond their labeled expiration date. By combining pharmacopeial standards with risk-based approaches like failure mode and effect analysis (FMEA), the study provides a comprehensive evaluation framework. This approach not only confirms the continued effectiveness of expired fluoroquinolone antibiotics but also underscores the potential waste reduction and cost-saving benefits. This could significantly contribute to addressing healthcare challenges in low-resource settings, promoting more efficient pharmaceutical resource utilization.

## Introduction

Medicines are compounds utilized to relieve illnesses; nevertheless, the timely administration of medications is essential for their efficacy, and wastage may result from factors such as excessive prescribing, expiration, or inadequate storage (1–3). Medications are costly, particularly, an Asian and African continents, where many people struggle with affordability (4). Drug waste in developing nations is high due to expiry, causing affordability and quality issues for many people (5). The expiration date of the drug plays a great role in retaining the drug substance of the Active pharmaceutical Ingredient (API) with regard to shelf life. The International Council for Harmonization of Technical Requirements for Pharmaceuticals for Human Use (ICH) outlines (5) the expiration date as a guarantee for the manufacturer to have their medicines in full potency and safety. In 2016, it was observed that pharmaceutical items stored under prescribed conditions remain viable and potent even after their expiration dates (6). The retaining ability of medicines potency may be depend on several essential parameters such; types of dosage, lots, preservatives, and storage conditions (7). The American Medical Association’s 2016 study found that solid dosage formulations can retain 90% of their potency for over 5 years if stored correctly in sealed packaging (8).

A study in the USA found that twelve expired drug products retained 90% of their labeled amounts, indicating that the expiration date does not guarantee potency beyond the established date (9). A study in the USA found that out of 122 evaluated drug products, 88% (2650 lots) retained their quality after expiration (10). In Africa, particularly in Ethiopia, weak drug regulations lead to expired drugs being disposed of without quality control laboratory testing, with a 5% expiration rate and an estimated 20 million ET (11). The study also showed that, an estimated of 8,723,541.62 ETB (42%) were solid forms found to be expired. Additionally, A 2022 study by Tura et al. (12) found that 48.81% of expired medicines in Arsi Zone health facilities were solid dosage forms. A study found no negative side effects from using expired medications from shelf life extension program stocks in developing countries, particularly Africa, despite the widely held belief that expired drugs are toxic (13). FDA advise using expired prescription drugs in supply chain disruptions, such as stockouts or outbreaks, as long as the manufacturer’s expiration date is followed (14).

Low-income countries like Ethiopia lack the safe, quality, and efficacious essential medicines (15). Moreover, large volumes of pharmaceutical products are expired in the health facilities in the setting due to numerous factors. The setting also experienced significant stock out of these medicines (16). Evaluating expired essential medications are pivotal to prevent financial losses and healthcare facility shortages. Although potency decreases post-expiration, extent of loss remains uncertain (17). Infectious diseases lead to a high distribution of antibiotics, which are at higher risk of expiring, posing bacterial proliferation risks, and ineffective disposal leading to treatment failure and antibiotic resistance (17). The safety of antibiotics, consumption statistics, clinical significance, and expired items in public health institutions underscore the unpredictable nature of medication safety based on past data (18). Recent study showed that over 90% of expired medicines are safe and effective even years after their expiration date, suggesting the potential for their recycling (19, 20). For example, a report from USA showed that from a total of 122 drugs evaluated, 88% of them past the quality evaluation test (10).

The FDA updates warnings on fluoroquinolone antibiotics, incorporating laboratory-based quality control studies, to ensure healthcare providers and patients are aware of their risks and benefits (21). The reasoning for selecting fluoroquinolone antibiotics in Ethiopian healthcare settings involved the assessment of ciprofloxacin and norfloxacin for efficacy post-expiration and during therapeutic administration. This evaluation was conducted by analyzing prescription trends, consumption rates, and disease prevalence (22–24). Apart, the average medicine waste rate of 4.87% in Jimma zone public hospitals during the fiscal years 2019/2020 and 2020/2021, amounting to $32,453.30, was significant (25). The fact that ciprofloxacin and norfloxacin were among the expired drugs indeed underscores the necessity for improvements in drug management practices. Ensuring optimal storage conditions is crucial for maintaining the efficacy and safety of pharmaceuticals. According to published evidence, health centers that meet more than 80% of the desirable storage condition criteria provide an optimal environment for drug storage, helping to prevent issues such as drug expiration and degradation (11, 26). Indeed, the study’s findings that inadequate storage conditions were particularly problematic for maintaining drug potency, including fluoroquinolones like ciprofloxacin and norfloxacin, in Western public health facilities of Ethiopia, highlight critical challenges in pharmaceutical management (11, 25).

The World Health Organization’s classification system for antibiotics is used to assess and track usage, with higher resistance potential antibiotic classes grouped under the Watch group in Ciprofloxacin and Norfloxacin tablets, emphasizing monitoring and stewardship efforts (27). The failure mode and effect analysis (FMEA) is critical for assessing risk and optimizing methods based on desirability parameters, thereby enabling the development of corrective and preventive actions (28). Failure Modes and Effects Analysis (FMEA) is a tool for conducting a systematic, proactive analysis of a process in which harm may occur in pharmaceutical environment (29). A study conducted in 2016 in Iran highlighted the significance of failure mode and effect analysis in identifying risk factors in pharmaceutical production, particularly in quality control and manufacturing processes (30).

The recent study conducted by Davido et al. (31) focused on the efficacy and safety of expired fluoroquinolone ciprofloxacin. The study examined 242 lots of ciprofloxacin and found that the content of the active substance in these expired lots maintained 100% efficacy. This suggests that ciprofloxacin retains its potency even after the expiration date under the conditions studied. This finding could have significant implications for the use and disposal of expired medications, particularly in resource-limited settings where access to essential medicines can be challenging. However, it is important to note that the study’s results should be interpreted with caution, and further research may be necessary to fully understand the safety profile and potential risks associated with using expired medications (31). The study conducted by Ramachandran (32) underscores that unused drugs may remain effective for more than 90% of lots for up to at least 5 years beyond their expiration date. However, it is important to note that the remaining 10% of these lots may become ineffective over time. This ineffectiveness poses a significant risk of unfavorable outcomes, particularly for critical infections where the efficacy of antimicrobial therapy is crucial (32).

The potential public health implications of confirming that expired fluoroquinolone antibiotics retain their potency and safety are substantial and multifaceted (32). It is essential to consider these consequences in order to guide public health policy and procedures. This data could lead to various important implications for public health, including: Savings in costs, enhanced availability of medications, decreased wastage, management of emergency stockpiles, changes in regulations and policies, patient adherence and confidence, concerns regarding antimicrobial resistance (AMR), and focus on research and development (33). Overall, the confirmation of the efficacy and safety of expired fluoroquinolone antibiotics could have far-reaching positive implications for public health, provided that appropriate measures are taken to manage and mitigate potential risks.

Even though expired medications were discovered in Ethiopian public institutions, there was insufficient evidence to assess the quality of the expired medications, specifically the 500 mg and 400 mg tablets of ciprofloxacin and norfloxacin. Taking these issues into consideration, the present study aims to explore the following key research questions to provide possible recommendations that can be tailored to enhance regulatory frameworks, establish quality control protocols for expired medications, and potentially guide policy revisions regarding their reuse. What is the quality status of the selected expired medications based on failure mode, effect analysis, and pharmacopeial specification tests?

## Materials and methods

### Study setting, and period

The expired samples were collected from public hospitals in the Jimma zone of the Oromia regional state, Ethiopia for the purpose of laboratory quality control test. The preliminary information collection regarding medicine expiration was started on 1/2/2022 G.C. The experimental quality control test was performed in the Jimma University Laboratory of Drug Quality (JuLaDQ) and started on 17 May 2022, and ended on 1 July 2022, G.C. Jimma is one of the zones in the Oromia regional state in southwest Ethiopia, around 350 kilometers from Addis Ababa, Ethiopia’s capital. According to the Jimma Zone Health Office report, the zone has twenty-six community pharmacies (*n* = 26), one hundred three (*n* = 103) drug stores, eleven (*n* = 11) hospitals (three general, one referral, and seven primaries), one hundred twenty-six (*n* = 126) health centers, and fifty-five (*n* = 55) health posts that provide healthcare services to a total of 986,957 people, according to the 2017 Central Statistics Agency report (13).

### Equipment’s

Various equipment, such as the Agilent 1200 Technologies HPLC system from Germany, the Tian Jin Optical Instruments RC-6D Dissolution Apparatus (Apparatus 2) from China, the Cecil Instruments UV/VIS spectrophotometer from the United Kingdom, the Mettler Toledo analytical balance from Switzerland, the AD 1020 pH meter, a 0.4 μm Whatman membrane filter, and different size volumetric flasks, were available at the Jimma University Laboratory of Drug Quality (JuLaDQ) for use in experimental quality control tests.

### Solvents/chemicals and reagents

Acetonitrile HPLC grade (Alpha Chemika, India), phosphoric acid (Reagent Chemical Service Ltd, Runcorn, United Kingdom), glacial acetic acid (Reagent Chemical Service Ltd), sodium hydroxide (UniChem chemical reagent, Hinton, United Kingdom), monobasic sodium phosphate (Sigma Aldrich, Hamburg, Germany), triethylamine HPLC grade (Sigma-Aldrich, Germany), hydrochloric acid (D.B. H Laboratory Supplies, England), and ultra-pure water (JuLaDQ) were utilized as the chemicals and reagents in the experiment. The ciprofloxacin USP working standard (potency/assay = 100.93%) was kindly provided by Ethiopian pharmaceutical manufacturing company Sh. Co. (EPHARM), and the Norfloxacin USP working standard was kindly provided by Cadila Pharmaceuticals (Ethiopia). PLC (potency = 98.94%) was used in the study.

### Study design, and approaches

The retrospective and prospective studies were utilized for collecting expired medicines for the purpose of quality control tests. The retrospective study technique was used for collecting information regarding expired medicines in the respective hospitals. The prospective method was used depending on the information gotten from the retrospective sample record and forwarding it to a laboratory quality control test. Accordingly, all relevant information concerning the expired medicines is relevant in the health sector to assure whether those expired medicines are within their established potency or not. The selection of the expired products was based on the FDA’s updated warnings and evidence-based decisions (21), consumption of data and diseases prevalence (22–24), and watch group should be established to monitor the category of organisms that possess a greater capacity to develop resistance. This group should consist of the most crucial agents among the Critically Important Antimicrobials for Human Medicine, along with antibiotics that are particularly susceptible to bacterial resistance (27). Two data collectors were assigned temporary recruitment depending on the number of samples collected.

### Sample size determination, and sampling techniques

No sampling technique was used in the hospital selection process; instead, all eleven hospitals located in the Zones were included (*n* = 11). The choice to focus solely on hospitals was made due to their ability to offer a diverse array of services, crucial data, and comprehensive records of expired medications. Out of the total number of hospitals, only nine (*n* = 9) were chosen for the study according to specific inclusion criteria (34). The Collection technique of expired Ciprofloxacin 500, and Norfloxacin 400 mg tablets was adopted from World Health organization quality evaluation sampling strategy (10). Accordingly, the overt sample collection technique was utilized in the selection of expired medicines from public hospitals. First, the research team communicated with the head office of the zone to get information regarding the existence of those expired medicines in the public hospitals. Then, the store manager and pharmacy head communicated the purpose of the study. Finally, all available expired Ciprofloxacin 500 mg and Norfloxacin 400 mg tables were collected. Accordingly, four and three batches of expired Ciprofloxacin 500 mg and Norfloxacin 400 mg tables were collected for quality evaluation parameters, respectively. A total of one hundred tablets were selected from each batch of expired medicines for experimental purposes.

Samples from the field could be collected with the help of a sample collection form that was structured to gather the following data for each sample: the nation in which the medicine was manufactured, the name of the medication, the dosage form, the package size, the batch or lot number, the brand or generic name, the claim or strength on the label, the date of manufacturing, and the date of expiration (Supplementary File 1).

The collected samples were transported to the Jimma University Laboratory of Drug Quality (JuLaDQ) and stored under the storage conditions specified on the label of the product until analysis. The detailed label information of different batches of Ciprofloxacin 500 mg and Norfloxacin 400 mg tablets collected from the respective hospital was attached as supplementary information (Supplementary File 2).

### Quality evaluation parameters, and risk analysis

Quality control measures including identification test, assay, and dissolution were taken into account in the current investigation to verify whether or not the medications taken by the expired candidates were within their prescribed potency. The United States Pharmacopeia’s procedure was followed in performing the quality control evaluation. In addition, risk analysis was carried out utilizing a number of high-quality risk management methods, such as FMEA (Failure Mode Effects Analysis), to estimate the risk related to the risks. Thus, in this study, the criticality of product quality features was assessed using FMEA. Using RPN, criticality was assessed based on assessments of the likelihood that a failure would occur (O), its severity (S), and the probability that it won’t be detected (D). The risk analysis was conducted as per Newton et al. (35).

### Identification test of ciprofloxacin 500 mg tablets

The retention time of the major peak in the chromatogram of the Assay preparation corresponds to that of the Standard preparation obtained as directed in the assay as per USP31/NF26 (36).

### Assay test for ciprofloxacin tablets

Preparation of diluents. Solution A: Firstly, a solution A was prepared by mixing 1.7 ml (0.025M) of phosphoric acid and distilled water in volumetric flask of 1,000 ml. Then, the solution was allowed to degassed and then filtered. Filtered and degassed mixture of phosphoric acid was then adjusted to a pH 2.0 ± 0.1 with triethylamine. Solution B: Solution B was prepared by mixing solution A and acetonitrile in the ratio of (13: 87). Finally, the filtered and degassed mixture of 0.025M phosphoric acid adjusted with triethylamine to a pH of 2.0 ± 0.1, and mixed with acetonitrile in the ratio of (87:13) was used as diluents. Solution C: Solution C was prepared by adjusting the volume of solution A to pH of 3.0 ± 0.1.

Mobile phase preparation. Mobile phase was prepared by mixing solution C and acetonitrile in the ratio of (87:13).

Preparation of ciprofloxacin working standard. About 0.5 g of working standard of ciprofloxacin was weighed and dissolved in filtered and degassed solution B in a 100 ml volumetric flask. Then made up to volume with a solution B. The prepared sample was filtered. Then, 4 ml of the filtrate was transferred in 100 ml volumetric flask and mixed up to volume with solution B. Finally, a solution containing 0.2 mg of ciprofloxacin per ml was obtained.

Chromatographic system. The liquid chromatogram equipped with a 278-nm detector and stainless-steel column (25 cm × 4.6 mm) packed with C18 was employed for the assay of ciprofloxacin tablets. Then, HPLC was adjusted with mobile phase maintained at 30 ± 1°C with the flow rate 1.5 ml per minute. Finally, equal volumes (10 μl) of the standard preparation and samples were injected into the chromatogram (36).

System suitability. The system suitability test involved the automatic injection of six replicates of 10 μm standard solutions into the HPLC. Subsequently, the column efficiency, calculated based on the ciprofloxacin working standard peak, must be equal to or greater than 2,500 theoretical plates. Additionally, the tailing factor for the ciprofloxacin peak should not exceed 2.0, and the relative standard deviation for replicate injections should be less than 1.5%. The System suitability criteria for High-Performance Liquid Chromatography (HPLC) assays are essential to ensure that the HPLC system is functioning properly before analyzing samples.

Sample solution preparation. An estimated amount of five tablets (*n* = 5) containing 500 mg of ciprofloxacin tablets from each batch were powdered and transferred to a 500 ml volumetric flask and then diluted with 400 ml of solution B. the prepared sample was sonicated for about 20 min. After the sample was sonicated, solution B was used to bring the sample solution up to volume. Then, the sample solution was filtered via a 0.4 μm what man membrane filter and 4 ml of filtrate was transferred to a 100 ml volumetric flask, where it was then brought up to volume with solution B. As a result, a concentration of ciprofloxacin of 0.2 mg per ml was obtained. Finally, 10 μl of the assay preparation was injected into the chromatograph. The percentage content of ciprofloxacin (in mg) was determined using peak areas of the sample and standard solutions using Equation (1).


(1) %ContentofCiprofloxacin500mgtablets=

(331.34 367.81)⁢(C⁢LD)⁢(r⁢ur⁢s)

Where,

331.34 and 367.81 were the molecular weights of ciprofloxacin and anhydrous ciprofloxacin hydrochloride, respectively, C = concentration, in mg/ml, of ciprofloxacin hydrochloride working standard in the standard preparation, calculated on the anhydrous basis, L = is the labeled quantity, in mg, of ciprofloxacin in each tablet, D = is the concentration, in mg/ml, of ciprofloxacin in the assay preparation based on the labeled quantity per tablet and the extent of dilution, ru = the ciprofloxacin peak areas obtained from the assay preparation, rs = the ciprofloxacin peak areas obtained from the standard preparation.

Acceptance criteria: Ciprofloxacin 500 mg tablets passes the test if content of ciprofloxacin tablets not lower than 90.0 percent and not more than 110.0 percent of the stated/labeled quantity, according to the USP method (36).

### Dissolution procedure for ciprofloxacin 500 mg tablets

Preparation of dissolution medium. The dissolution test was carried out on the tablets as per the USP dissolution apparatus II (paddle method). First dissolution medium was prepared by mixing 0.84 ml (0.01N) of hydrochloric acid with distilled water in 1,000 ml volumetric flask. Then, dissolution medium 900 ml of 0.01N hydrochloric acid was prepared.

Preparation of the calibration curve for dissolution study. In a dissolution study for Norfloxacin tablets, preparing a calibration curve is crucial for quantifying the amount of drug released over time. The calibration curve prepared for the dissolution study of Norfloxacin tablets helps in quantifying the drug release from the tablets. It indicates the relationship between the concentration of Norfloxacin and the HPLC detector response, ensuring accurate and reliable measurement of the drug in the dissolution samples. About 13.88 mg of ciprofloxacin working standard was weighed and transferred to 25 ml volumetric flask and mixed with dissolution medium up to the volume. Then the sample solution was filtered after that. The filtrate of 4, 4.5, 5, 5.5, and 6 ml were transferred to 200 ml volumetric flask and mixed with the dissolution medium up to the volume to prepare concentrations of 11, 12.5, 13.88, 15.27 and 16.65 μg/ml, respectively. In a UV-visible spectrophotometer, the absorbance of the prepared working standard solutions was measured at 276 nm. Then, calibration curve was then determined by plotting the concentration of ciprofloxacin working standard vs. absorbance reading from UV-visible spectrophotometer. At the end, the calibration curve of ciprofloxacin working standard was plotted against reading absorbance obtained from spectrophotometer using Equation (2). From the calibration curve the concentration of analytes were determined.


(2) Y=mx+c

Where,

Y = Absorbance reading of the UV-visible spectrophotometer, m = Slope of the straight line, c = intercept on the AUC (y axis), and x = concentration of analyte.

Ciprofloxacin tablets sample preparation. The dissolution test was conducted according to the USP monograph on six tablets of each batch using dissolution tester having six vessels and equipped with rotary paddles (USP Apparatus 2) operated at 50 revolutions per minutes. Exactly six tablets per batch were suspended in each vessel containing 900 ml of 0.01N of Hydrochloric acid solution maintained at 37 ± 0.5°C and the dissolution process was monitored over 60 min. Then, 10 ml of sample was withdrawn at 15, 30, 45 and 60 min from all six-dissolution vessel at different interval with 10 ml of fresh dissolution medium replacement maintained at the same temperature of dissolution medium for every withdrawal.

Then the withdrawn sample solution from all six-dissolution vessels were filtered using 0.4 μm what man filter paper. Then 5 ml filtrate of each sample solution was transferred in to 200 ml volumetric flak and made up to the volume with the dissolution medium. The absorbance of the diluted solution was measured using UV/Visible spectroscopy at 276 nm using 0.01N HCL as blank. The average absorbance readings obtained from six dissolution vessels for each sample solution was used to calculate the amount of released ciprofloxacin APIs at a given time point. Percentage release of ciprofloxacin hydrochloride tablet was determined from the absorbance of the sample using the equation created from calibration curve. The USP stated that amount of ciprofloxacin hydrochloride tablet equivalent to not less than 80% of the labeled amount of ciprofloxacin is dissolved in 30 min (36).

### Assay study of the Norfloxacin 400 mg tablets

#### Identification test of norfloxacin tablet

The identification test was carried out by comparing the retention time of the major peak in the chromatogram of Norfloxacin 400 mg tablet with that of the Norfloxacin working standard obtained directly from assay test results (37).

#### Assay preparation of norfloxacin

Chromatographic system. The liquid chromatogram equipped with a 275-nm detector and a 3.9 mm × 30- cm column packed with a stationary phase of 5-μm particle size and operates at 40 ± 1.00C was used. The flow rate used for preconditioning and assay was maintained 0.5 and 2 mL/minute, respectively. Finally, 10 μl equal volumes of the standard preparation and sample solution were injected into the chromatogram.

System suitability. Firstly, about 0.01 M monobasic sodium phosphate was prepared by dissolving 1.5 mg of monobasic sodium phosphate with distilled water in 1,000 ml volumetric flask. Then it was adjusted to a pH of 4.0 with phosphoric acid and degassed. Following, column preconditioning was performed for 8 h with the prepared solution. After completion of column preconditioning, six replicate injections of 10 μm of standard solutions were injected automatically to the HPLC. Six replicate injections were used to determine system suitability for relative standard deviation (< 2%), and tailing factor not more than 2 (37).

Preparation of diluents. A solution containing Phosphoric acid solution and distilled water was prepared in the ratio of (1 in 1,000). This solution was then degassed and filtered.

Mobile phase preparation. Mobile phase was prepared by mixing diluents and acetonitrile in the ratio of (850:150), respectively.

Working standard preparation. An accurately weighed 20 mg of USP Norfloxacin working standard was dissolved in mobile phase in 100 ml volumetric flask. Finally, concentration of norfloxacin working standard 0.2 mg/ml was obtained.

Norfloxacin tablets sample preparation. Twenty (*n* = 20) Norfloxacin tablets from each batch were weighed and finely powdered. An amount of powder equivalent to 100 mg of Norfloxacin was transferred to a 200 ml volumetric flask. Then, an estimated 80 ml of mobile phase poured and blended. Then, the solution was sonicated for 10 min and made up to the volume with diluents and then mixed. From the mixed solution about 10 ml of solution was withdrawn and transferred to a 25 ml of volumetric flask. Then, the transferred solution was diluted to a required volume with the mobile phase, and stirred. Finally, the solution was filtered by Whatman filter paper with a porosity of 0.4 μm. Then, 10 μl of the filtered assay solutions were injected automatically to the chromatography. The percentage content of norfloxacin (in mg) was determined using peak areas of the sample and standard solutions using Equation (3).


(3) $$\text{\%content of API = 500 }\left(\frac{\text{ru}}{\text{rs}}\right)$$

Where;

C is the concentration, in mg/mL of norfloxacin reference standard in the standard preparation and ru and rs are norfloxacin peak responses obtained from the assay preparation and the standard preparation, respectively.

Acceptance criteria. Norfloxacin tablets should contain not less than 90%, and not more than 110% of the stated/labeled amount, according to the USP method (37).

### Dissolution procedure study

Dissolution medium preparation. First, 1 ml of 50% (w/w) solution of sodium hydroxide was prepared by dissolving 5 mg of sodium to 10 ml of distilled water. Then 2.86 ml of glacial acetic acid was mixed with 1.0 ml of a 50% (w/w) solution of sodium hydroxide to 900 ml of water in a 1,000 ml volumetric flask and made up to volume with distilled water. Therefore, 750 mL acetate buffer maintained at 37°C ± 0.5°c and adjusted with glacial acetic acid to a pH of 4.0 was used as dissolution medium.

Preparation of calibration curve for dissolution test. The calibration curve prepared for the dissolution study of Norfloxacin tablets was essential for ensuring the accuracy and precision of the analytical method. By measuring the absorbance of standard solutions at known concentrations and plotting these values to obtain a linear relationship, the calibration curve provided a reliable means to quantify Norfloxacin in dissolution samples. This ensured that the dissolution study results were scientifically sound and reproducible, which is critical for assessing the quality and efficacy of Norfloxacin tablets.

A stock solution was prepared by dissolving 10 mg of norfloxacin USP working standard in 100 ml of acetate buffer. Then six concentration levels 1, 2, 3, 4, 5, 6 μg/ml was prepared from the stock solution by diluting 2, 4, 6, 8, 10, and 12 ml of the stock solution in 200 ml volumetric flask and diluted with acetate buffer to the volume. Then, their absorbance was determined spectrophotometrically. Then concentrations of norfloxacin were plotted against their absorbance to obtain the calibration curve.

Sample preparation. The dissolution test was conducted according to the USP monograph on six tablets of each batch using dissolution tester equipped with rotary paddles (USP Apparatus 2) operated at 50 revolutions per minute. Exactly six tablets from each batch were suspended in each vessel containing 750 ml acetate buffer solution adjusted to pH of 4.0 and maintained at 37°C ± 0.5°c. The dissolution process was monitored over 60 min. Then, 5 ml of dissolution medium was sampled at 15, 30, 45, and 60 min with replacement of 5 ml of fresh dissolution medium for every withdrawal. Then 5 ml of sampled solution was filtered and 1 ml of the filtrate was transferred to 200 ml volumetric flask and mixed with acetate buffer solution to the volume. The corresponding absorbance reading of diluted filtrates was taken by UV-Vis spectrophotometer at a wavelength of 278 nm. The average absorbance readings obtained from six dissolution vessels for each sample solution was used to calculate the amount of released norfloxacin at a given time point. Percentage release of norfloxacin tablet was determined from the absorbance of the sample using the equation created from calibration curve. According to USP, an amount of norfloxacin equivalent to not less than 80% of the labeled amount of norfloxacin is dissolved in 30 min (37).

### Risk analysis

Risk analysis was conducted according to the method described in the Bozdag et al. (38) literature. To determine the criticality of product quality features, failure modes effect analysis (FMEA) was used. The risk priority number (RPN) was calculated using Equation (4).


(4) Risk⁢probaility⁢number=O⁢x⁢S⁢xD

The equation took into account the failure’s occurrence (O), severity (S), and detection (D). The scales used for scoring severity, occurrence and detectability which was taken from reference manual developed by failure mode and effects analysis (FMEA) teams at Chrysler, Ford and General Motors (24), and are presented in Tables 1–3, respectively.

**TABLE 1:** Evaluation criteria and ranking system for the occurrence of failure.

Probability of failure	Possible failure rates	Rank
Extremely high: failure almost inevitable	≥ 1 in 2	10
very high	1 in 3	9
Repeated failures	1 in 8	8
High	1 in 20	7
Moderately high	1 in 80	6
Moderate	1 in 400	5
Relatively low	1 in 2,000	4
Low	1 in 15,000	3
Remote	1 in 150,000	2
Nearly impossible	≤ 15,000,000	1

**TABLE 2:** Evaluation criteria and ranking system for the severity of effects.

Effect	Criteria: severity of effect	Rank
Hazardous	Failure is hazardous, and occurs without warning. It suspends operation of the system	10
Serious	Failure involves hazardous outcomes and/or noncompliance with government regulations or standards	9
Extreme	Product is inoperable with loss of primary function. The system is inoperable	8
Major	Product performance is severely affected but functions. The system may not operate	7
Significant	Product performance is degraded. Comfort or convince functions may not operate	6
Moderate	Moderate effect on product performance. The product requires repair	5
Low	Small effect on product performance. The product does not require repair	4
Minor	Minor effect on product or system performance	3
Very minor	Very minor effect on product or system performance	2
No effect	None	1

**TABLE 3:** Evaluation criteria and ranking system for the detection of a cause of failure.

Detection	Design control does not detect a potential cause of failure or subsequent failure mode; or there is no design control	Rank
Absolute uncertainty	Design control does not detect a potential cause of failure or subsequent failure mode; or there is no design control	10
Very remote	Very remote chance the design control will detect a potential cause of failure or subsequent failure mode	9
Remote	Remote chance the design control will detect a potential cause of failure or subsequent failure mode	8
Very low	Very low chance the design control will detect a potential cause of failure or subsequent failure mode	7
Low	Low chance the design control will detect a potential cause of failure or subsequent failure mode	6
Moderate	Moderate chance the design control will detect a potential cause of failure or subsequent failure mode	5
Moderately high	Moderately high chance the design control will detect a potential cause of failure or subsequent failure mode	4
High	High chance the design control will detect a potential cause of failure or subsequent failure mode	3
Very high	Very high chance the design control will detect a potential cause of failure or subsequent failure mode	2
Almost certain	Design control will almost certainly detect a potential cause of failure or subsequent failure mode	1

For occurrence, literature was reviewed for the ciprofloxacin tablets and there was no study conducted for the expired norfloxacin. Therefore, pharmacopeial quality evaluation of norfloxacin in this study showed, there was the least specification limit (90%) obtained for assay. The study conducted by the US Department of Defense on expired ciprofloxacin tablets from (242 batches) (10) and Israel ministry of health from (15 batches) (29) revealed that all of tested samples were found in pharmacopeial specification for quality evaluation.

To establish a severity score, the probable impact of each failure mode was considered (Table 2) including hazardous, serious, extreme, major, significant, moderate, low, minor, very minor, no effect.

In detection of both drugs relatively advanced equipment (HPLC), advanced knowledge and skills is needed for identity and assay than for dissolution. Hence, high rate of detection was given to identity and assay than dissolution (Table 3).

Based on the information obtained from ciprofloxacin 500 mg tablets and norfloxacin 400 mg tablets the following numerical values were assigned to compute for risk probability number (Table 4).

**TABLE 4:** Assigned scores for factors of the evaluated failure modes of Ciprofloxacin and Norfloxacin tablets.

Ciprofloxacin-identity		
Severity	Occurrence	Detectability
10	2	7
**Ciprofloxacin-assay**		
Severity	Occurrence	Detectability
9	3	7
**Ciprofloxacin-dissolution**		
Severity	Occurrence	Detectability
5	5	4
**Norfloxacin-identity**		
Severity	Occurrence	Detectability
10	2	7
**Norfloxacin-assay**		
Severity	Occurrence	Detectability
9	6	7
**Norfloxacin-dissolution**		
Severity	Occurrence	Detectability
5	5	4

Desirability Function. This parameters is significant in assessing the overall quality of examined products because it provides a comprehensive, objective, and quantifiable measure of product performance. By integrating multiple quality attributes into a single score, it aids in optimizing processes, balancing trade-offs, and making informed decisions to achieve high-quality products.

Based on the method given in Derringer G and Suich R. literature, the desirability function was also used to assess the quality of the examined product (39). The overall desirability function (D) value which is the geometric mean of the individual desirability (di) indicates the product quality. The highest global desirability value represents the product with the highest quality. The D value was calculated using Equation (5).


(5) $$\sqrt[{n}]{\prod_{i=1}^{n}d\,i*p\,i}$$

Where pi is the weight or relative importance assigned to the response, *n* is the product quality attributes evaluated, di is an individual desirability.

Three product quality attributes (identification, assay, and dissolution) were examined in this investigation and hence *n* = 3 was used in the overall evaluation of the ciprofloxacin and norfloxacin tablet samples. A desirability function’s main goal is to provide a single ball-mark figure that is a composite number that reflects various responses (40). This is accomplished by converting the value of each property/response into a numeric score ranging from 0 (one or more product features are absolutely unacceptable) to 1 (all product characteristics are on target) depending on the property/appropriateness response’s (or desirability).

Based on a psychophysical scale and the findings of the FMEA quality evaluation, individual desirability functions were constructed for each of the quality characteristics. The conversion of the quantity value of a certain quality indicator into an assessment of the acceptability (preference) of a certain condition of an examined subject (pharmacopeia quality of the two medicines) is classified by the desirability function, which has values in the range (0–1). A psychophysical scale of Harrington is chosen as one of the specific techniques to implement the desirability function for the corresponding estimation. The scale was used to determine the relationship between physical and psychological parameters. Physical parameters are all numeric desirability values (0–1) of measurable characteristics/quality features, whereas psychological parameters are solely subjective assessments of a researcher (e.g., excellent, good, acceptable, low, bad) to describe degree of satisfaction.

Modified psychophysical five-interval scale of Harrington’s desirability function (40) was constructed and used as a tool for quality judgment; i.e., qualitative assessments “bad,” “low,” “acceptable,” “good,” and “excellent” which correspond to numeric intervals of 0.00–0.37, 0.37–0.70, 0.70–0.80, 0.80–0.90, and 0.90–1.00, respectively. A two-sided desirability function was utilized for assay and dissolution, while a one-sided desire function was used for identity. Because the absence of API is thought to be clinically undesirable, *d* = 0 was assigned. While *d* = 1 was assigned to 100%lc, which is thought to be the optimum desirability. Because the assay of both ciprofloxacin, and norfloxacin tablet APIs in tablet should be between 90 and 110%lc and the psychophysical Harrington’s scale specifies desirability range from 0.70 to 1.00 to be good, *d* = 0.7 was assigned for assay values of 90 and 110%lc and *d* = 0.3 was assigned for 70% and 130%lc. While *d* = 0.01 was assigned for 50% and 150%lc. The individual desirability function for assay was then defined as distinct linear sections of different slopes in the range of 100 to 90%lc (slope = 0.03), 90 to 70%lc (slope = 0.02) and from 70 to 50%lc (slope = 0.01) as presented in Supplementary Figure 1. Similar but negative slopes were used for the assay greater than 100% (Supplementary Figure 1).

Since both medicines were in class IV BCS (41) dissolution is important quality evaluation parameters as well. Hence, the percent drug release was taken into account. Both ciprofloxacin and norfloxacin tablets should release 80% of their active ingredient within 30 min, according to USP acceptance standards. As a result, 100% drug release was assigned to *d* = 1, whereas 80 and 110% drug release was assigned to *d* = 0.7 for both medicines. Furthermore, 60 and 130% drug release were assigned *d* = 0.3, while 40 and 150% drug release were assigned *d* = 0.01. The individual desirability function for dissolution was then defined as distinct linear sections of different slopes in the range of 100 to 80%lc (slope = 0.015), 60 to 80%lc (slope = 0.02) and from 40 to 60%lc (slope = 0.01) as can attached in Supplementary Figure 2. Similar but negative slopes were used for dissolution greater than 100% (Supplementary Figure 2).

### Data processing and analysis

The experimental part of the finding was analyzed using Microsoft Excel 2010, and Statistical Package for Social Sciences software version 22. The Identity, assay, and dissolution test was carried out according to method specified in USP in triplicate and data were expressed as Mean ± RSD . A more detailed statistical data analysis was done based on the fixed effects model with different response variables (product quality attributes). FMEA was used to assess the criticality of the quality risks associated with each quality attribute and Derringer’s desirability function was applied to evaluate quality of the products. The risk probability number (RPN) values were computed after scoring assigned to each failure mode through multiplication of the total variables to obtain the total risk probability number. Finally, the experimental findings were displayed through graphs, and tables depending on the nature of the variables under consideration.

## Results

### Quality control, and risk analysis results

Tested product information. In the actual study, a total of four batches of ciprofloxacin 500 mg and three batches of norfloxacin 400 mg tablets were involved in the experimental work. The codes for the batches were given by the quality control research team of Jimma University in the school of pharmacy, which were coded as C-001, C-002, C-003, and C-004 for Ciprofloxacin 500 mg tablets, and N-005, N-006, and N-007 for Norfloxacin 400 mg tablets, respectively. All samples from each batch were studied through the evaluation of identification, dissolution, and assay.

### Assay study of ciprofloxacin hydrochloride tablet

System suitability. The system’s suitability was checked by six replicate injections of working standards solutions. As can be seen in Table 5, the system was suitable for laboratory analysis of identification, assay, and dissolution tests.

**TABLE 5:** System suitability test result for chromatographic method of assay of ciprofloxacin tablet.

System suitability test	Value observed	USP-NF limit	Compliance
Column efficiency	6,598.26	Not less than 2,500	Compliant
Tailing factor	1.1	Not greater than 2	Compliant
Relative standard deviation (RSD)	0.138	Not greater than 1.5	Compliant

USP–NF; United States Pharmacopeia–National Formulary.

Identification test study of ciprofloxacin. The confirmation of ciprofloxacin’s identity in the tablets was established through HPLC in accordance with the specifications outlined by the USP. As per the latest validation guideline from SANTE/SANCO, the retention times of analytes in both the sample and standard solution should not deviate by more than 0.1 min, which is a modification of the identity test specifications stated in the European Commission decision (42). The European Commission Decision 2002/657/EC specify a tolerance criterion for an analyte retention time relative to the standard substance retention time. The relative retention time of an analyte in a standard solution must correspond to that in a sample within a tolerance of 2.5% (Supplementary Figure 3). In the study, the retention time deviation ranged from 0.01 to 0.04 min, which satisfied a standard solution that must not differ more than by 0.1 min and be within the tolerance limit of 2.5% of the sample retention time (Table 6). Thus, all samples examined passed the identity test.

**TABLE 6:** Identification test result of ciprofloxacin working standard and samples.

Sample code	Retention time of samples (*n* = 2)	Retention time of Working standard S (*n* = 6)	Deviation in minutes
C-001	11.45	11.46	0.01
C-002	11.44		0.02
C-003	11.43		0.03
C-004	11.42		0.04

Assay of ciprofloxacin hydrochloride tablets. Volumes of 10 μl of assay preparation were injected twice from each batch solution into the HPLC. The average peak area was used to calculate the percentage content present in the ciprofloxacin tablets. Ciprofloxacin tablets should contain not less than 90% and not more than 110% of the stated amount as per USP (Table 7). All the batches of tested ciprofloxacin tablets passed as per USP specifications. The lowest drug content was obtained for batches C-004 (100.87), while the highest percentage drug content was obtained for batches C-002 (104.69).

**TABLE 7:** Percentage content of ciprofloxacin tablets.

Sample code	% API (mean R*SD*)	USP specifications	Compliance
C-001	103.35 ± 0.43	90–110%	✓
C-002	104.69 ± 0.21		✓
C-003	101.23 ± 0.38		✓
C-004	100.87 ± 0.01		✓

RSD, relative standard deviation; API, active pharmaceutical ingredient; USP, United States Pharmacopeia, ✓, complaint.

### Dissolution study of ciprofloxacin

Calibration curve for ciprofloxacin working standard. Five points standard concentrations ranged from 11, 12.5, 13.88, 15.27, and 16.65 μg/ml were prepared to construct the standard calibration curve as per Food and Drug Adminstration method verification and validation guideline (36). As indicated from the result of the calibration curve (Supplementary Figure 4) there was a strong linear relationship (*r* = 0.998) between the concentration of the working standard and the absorbance values.

Dissolution profile of ciprofloxacin tablets. The dissolution test result revealed that samples C-002 was release its drug content less than 80% at 30-min single point while the other three samples were releasing their drug content greater than 80% at 30 min at (Table 8) single point as per USP pharmacopeia specification (36).

**TABLE 8:** Percentage release of tested ciprofloxacin hydrochloride tablet.

Sampling time (minute)	Sample code			
	**001**	**002**	**003**	**004**
15	89.32	65.09	60.57	72.83
30	94.29	78.67	87.25	83.88
45	95.23	83.56	90.57	94.27
60	98.46	89.40	97.57	97.99

### Assay study of norfloxacin 400 mg tablet

Chromatographic system. The System is suitable in respect to the suitability tests as per requirements of United States Pharmacopeia. The results of system suitability test of norfloxacin working standard were listed in Table 9.

**TABLE 9:** System suitability test result for chromatographic method of assay of norfloxacin tablet.

System suitability test	Value observed	USP-NF limit	Compliance
Tailing factor	1.02	Less than 2	Compliant
RSD	0.068	Less than 2	Compliant

RSD, relative standard deviation; USP-NF, United States National Formulary.

Assay test result for norfloxacin tablets. According to the assay result, batch N-005 had the minimum requirements percentage content of active ingredient (90%) and batch N-007 had a highest percentage content of active ingredient of 99.55%. All batches of norfloxacin tablets compliant with requirements percentage content of active ingredient (Table 10) set by pharmacopeial specifications which is within (100 ± 10%) of label claim as described in USP pharmacopeia (37).

**TABLE 10:** Result for assay of norfloxacin tablet.

Sample code	% API (*n* = 2) (M*ean* = R*SD*)	USP specification	Compliance
N-005	90 ± 0.32	90–110%	✓
N-006	94.12 ± 0.05		✓
N-007	99.55 ± 0.18		✓

RSD, relative standard deviation.

Identification test for norfloxacin tablets. The identity of norfloxacin in the tablets was confirmed by HPLC as per USP specifications. According to the most recent SANTE/SANCO validation guideline, retention times of an analyte in a sample and in a standard solution must not differ more than by 0.1 min, modified from the European Commission decision specification for identity tests (42). The retention time of major peak of the sample solutions of all batches of norfloxacin tablets were correspond to that of the standard solution (Table 11), as obtained in the assay (Supplementary Figure 5). Thus, all samples of norfloxacin tablets examined for the identity of active ingredient passed the identification test of USP specifications (37).

**TABLE 11:** Identification test result of norfloxacin working standard and samples.

Sample code	Retention time of samples (*n* = 2)	Retention time of WS (*n* = 6)	Deviation in minutes
N-005	0.631	0.628	0.003
N-006	0.629		0.001
N-007	0.6275		0.0005

WS, working standard.

### Dissolution study of norfloxacin 400 mg tablet

Calibration curve for norfloxacin working standard. Six concentration levels 1, 2, 3, 4, 5, 6 μg/ml of working standard were prepared to construct standard calibration curve (37). As indicated from the result of the calibration curve there was a strong linear relationship (*r* = 0.997) between the concentration of the working standard and the absorbance values (Supplementary Figure 6).

Dissolution profile of norfloxacin tablet. As obtained from dissolution test result all samples were release greater than 80% of its drug content at 30-min single point which showed that all samples comply with USP specification stated that norfloxacin release its content should not less than 80% within 30 min. Drug product Code N-005 (101.84%) had the highest percentage of drug release, while drug product code N-007 (93.84%) had the least amount of drug release at a single time point (Table 12).

**TABLE 12:** Percentage release of tested norfloxacin tablet.

Sampling time (minute)	Sample code		
	N-005	N-006	N-007
15	78.35	88.26	81.76
30	101.84	94.65	93.84
45	103.64	98.58	96.76
60	104.37	100.10	101.79

In general, the pharmacopeia quality control test depicted that, about 100% (7/7) expired samples passed pharmacopeia quality specifications for identity and assay tests. However, of the seven expired brands, about 14.3% (1/7) of the sample (Code-002) was unable to release its API content within the USP criteria of 30 min.

### Risk analysis result

The failure modes of identity (no active intended ingredients in the sample) or mislabeling (incorrect, inadequate, or incomplete identification), assay (under-dose and overdose), and dissolution (inability to sufficiently dissolve within a specified time period) were considered in the determination of the risk probability number.

In addition to this, the failure effects of identity [death due to untreated diseases, toxicity, treatment failure, assay (toxicity due to overdose), and dissolution (treatment failure and poor absorption and bioavailability)] were also considered. To calculate the risk probability number, scores assigned to each failure mode were multiplied together to obtain the total risk probability number.

As can be seen in Table 13, the critical quality attributes were subjected to failure mode and effect analysis (FMEA). Assay was the most critical quality attributed to ciprofloxacin tablets (RPN = 189), followed by identity (RPN = 100). Assay was also the most critical quality attribute (RPN = 378), followed by identity (RPN = 100) for Norfloxacin tablets. The finding confirmed that assay and identity were the most critical quality attributes to the product of ciprofloxacin 500 mg and Norfloxacin 400 mg tablets, according to the risk probability number computed method.

**TABLE 13:** Failure mode and effect analysis for ciprofloxacin tablet quality attributes.

Product name	CQA	Severity	Occurrence	Detectability	Risk probability number
Cipro*	Identity	10	2	7	140
	Assay	9	3	7	189*
	Dissolution	5	5	4	100
Nor*	Identity	10	2	7	140
	Assay	9	6	7	378*
	Dissolution	5	5	4	100

Cipro*, ciprofloxacin 500 mg tablets; Nor*, Norfloxacin 400 mg tablets; CQA, critical quality attributes; *stands for the most critical quality attributes.

### Desirability function test result

The results of individual desirability values (di) for ciprofloxacin and norfloxacin tablets are presented in Supplementary File 3. A global D-value of investigated products was calculated using d-functions (Equation 4) of quality attributes (i.e., identity, assay and dissolution). According to RPN value, which is the numeric assessment of risk assigned to each quality parameter, *p*-value of “3” was assigned for assay since quality risk associated to it was found to be more important. Similarly, *p*-value of “2” was assigned for identity since the risk associated with identity was of more concern than dissolution and *p*-value of “1” was assigned for dissolution in both drugs. For each medicine analyzed for 3 quality attributes (assay, dissolution and identity), a global D-value of the tested products was finally calculated using d-functions (Equation 4), and the quality of the product were evaluated using the psychophysical Harrington’s scale of quality (Table 14).

**TABLE 14:** Global D-value of investigated ciprofloxacin, and norfloxacin tablets.

Codes	d-assay	d-dissolution	d-identity	Product	D-global	Quality, linguistic evaluation
C-001	0.90	0.91	1	0.662	0.872	Good
C-002	0.86	0.67	1	0.425	0.75	Acceptable
C-003	0.96	0.81	1	0.724	0.898	Good
C-004	0.97	0.76	1	0.702	0.889	Good
N-005	0.70	0.83	1	0.285	0.7	Acceptable
N-006	0.82	0.92	1	0.507	0.80	Good
N-007	0.99	0.91	1	0.883	0.96	Excellent

As can be seen in Table 15, the desirability function showed that 75% (3/4) of ciprofloxacin products were of good quality, and 25% (1) were found to be of acceptable quality. Additionally, the desirability function of norfloxacin tablets was found to be excellent, good, and acceptable; both accounted for 1 (33.3%). Therefore, all-expired medicines were within the risk-based desirability specification (0.70–1.00). Harrington’s scale is used to evaluate the desirability of various quality attributes and can be modified to suit specific needs in pharmaceutical assessments. The scale typically ranges from “poor” to “excellent” and is used to assess the overall quality of products based on different attributes.

**TABLE 15:** Modified psychophysical Harrington’s scale of quality and results of risk-based desirability function approach for Ciprofloxacin, and Norfloxacin tablets.

Product name	Intervals in global desirability (D-global)	Quality, descriptive evaluation	Number of products in each quality scale (percentage)
Ciprofloxacin	0.90–1.00	Excellent	–
	0.80–0.90	Good	3 (75%)
	0.70–0.80	Acceptable	1 (25%)
	0.37–0.70	Low	–
	0.00–0.37	Bad	–
Norfloxacin	0.90–1.00	Excellent	1 (33.3%)
	0.80–0.90	Good	1 (33.3%)
	0.70–0.80	Acceptable	1 (33.3%)
	0.37–0.70	Low	–
	0.00–0.37	Bad	–

## Discussion

Despite progress made, numerous people still face challenges in accessing essential medications. This is due to obstacles hindering developing nations from acquiring top-notch, cost-effective, and prompt pharmaceuticals. Such barriers not only compromise human dignity but also the core of all human rights, specifically the rights to life, health, and progress. Moreover, the issue of accessing quality medicines is a significant global public health concern, particularly in low- and middle-income countries (43). As per published literature, parallel to the limited accessibility of essential medicines, a high number of medicines expired in Ethiopian public institutions as they were in other developing countries around the world (11, 12). Despite the presence of expired medicines in public institutions (11), there is no evidence for a reanalysis study to confirm the potency of the expired drugs and the risk factors for critical quality attributes. Therefore, the study attempted to evaluate the quality of expired medicines and their risk for critical quality attributes for further targeted pharmaceutical quality intervention.

The quality of medicines is essential for efficient disease management. To ensure that good quality medicines are available to patients in their countries, national medicines regulatory authorities can apply various regulatory instruments. From the regulatory instruments, post-marketing surveillance activities through quality control testing plays critical for quality control evidence (44). Expired medications, in particular, are thought to lose their efficacy after the stated shelf-life (45). An assessment was carried out to determine if expired medications underwent a potency identification test in accordance with USP quality standards to verify the presence of the relevant active ingredient. The results showed that all batches of ciprofloxacin and norfloxacin tablets met the USP criteria for identification tests. This confirmed that the medications contained the necessary active ingredients for therapeutic purposes, ensuring their effectiveness. It is crucial for medicines to contain the approved quantity of API to achieve the desired therapeutic outcomes. Research has shown that using antibiotics with inadequate levels of active pharmaceutical ingredients can result in severe consequences such as drug resistance, treatment failure, and increased treatment costs (15). Incorrect dose adjustment leads to undesirable consequences such as increased morbidity, mortality, treatment costs, and hospital stay days (46). All batches of ciprofloxacin and norfloxacin tablets that were tested after their expiration dates met the USP specification limit for the indicated assay on the Pharmacopeia (90–110%). Consequently, the research demonstrated that relying solely on the expiration date as a validation of the drugs’ potency is not entirely reliable. Therefore, it is crucial to have post-market quality surveillance backed by laboratory quality control for reliable evidence.

This research reinforces prior findings that drugs can keep their API content after their expiration dates have passed. This is supported by a study undertaken by the united states department of defense, in which the FDA assessed different batches of drugs in their supply (29). From tested expired products, nearly 90% (122) of different medicine products studied met the requirements for extensions (47). The US Department of Defense conducted a shelf-life extension program, during which they assessed 242 batches of Ciprofloxacin. Remarkably, all of these batches successfully passed quality evaluations. Additionally, a separate study discovered that expired medicines, which were stored at the recommended temperature by the manufacturer, retained at least 98% of their indicated drug content for a significant duration of 18–170 months beyond their expiration dates (39).

The process by which an active ingredient dissolves in a solvent to produce a solution is known as dissolution and the test is used to determine how long it takes for medicine in an oral solid dosage form to dissolve under specific conditions (48). If the medicine fails to release the required amount at a specific time, it will not dissolve properly, resulting in reduced bioavailability. According to USP specifications, the ciprofloxacin and norfloxacin tablets in the study should release at least 80% of the indicated dosage within 30 min. With the exception of C-002 (78.67%), all the tested medicines met the USP single-point dissolution test requirement. The variation in dissolution properties at the 30-min mark could be attributed to differences in excipients used in the production of the pharmaceuticals, as well as other manufacturing-related activities and storage conditions (49).

A desirability function test based on risk, in addition to the pharmacopeial test, was used to evaluate medication quality. FMEA is a recognized tool for assessing key components to prevent failure and improve system reliability (50). In this study, the FMEA method was used to assess the risk of failure modes (identity, assay, or dissolution). The findings showed that assay was the most important quality attribute for ciprofloxacin tablets (RPN = 189), followed by identity (RPN = 140) and dissolution (RPN = 100). Similarly, for norfloxacin tablets, assay (RPN = 378) was found to be the most critical quality attribute, followed by identity (RPN = 140) and dissolution (RPN = 100). It can be inferred from this information that the risk of quality issues related to the assay is comparatively higher. Therefore, the optimization of active ingredients in the final formulation is of utmost importance in preventing quality failures in pharmaceutical products. A similar study conducted in Jimma also revealed that assay (RPN = 512) and dissolution (RPN = 336) were identified as the most critical quality attributes (51).

If any product characteristics go beyond desired limits, its quality is deemed unsatisfactory. In this study, product quality was assessed by comparing calculated D values with predetermined numeric ranges of 0 to 1. A modified version of Harrington’s scale of quality, corresponding to specific numeric intervals, was used to qualitatively evaluate the products (low, bad, good, acceptable, excellent) (40). Based on the results obtained from the risk-based desirability approach of quality evaluation and considering ciprofloxacin and norfloxacin APIs content and percentage of drug release, 100% of the quality of the investigated products lies within the acceptable range (0.70–1.00). As a result, utilizing a risk-based desirability method gives clinically more important quality attributes greater weight, allowing for more reliable decisions based on clinically more important quality attributes. Furthermore, the risk-based desirability method to quality assessment penalizes marginal out-of-specification drugs less strongly, which is thought to save money in a resource-constrained society (51).

Comparing the two quality evaluation methods, the pharmacopeia methods inform us that about 100% (7/7) expired samples passed pharmacopeia quality specifications for identity and assay tests. However, of the seven expired brands, about 14.3% (1/7) of the sample (Code-002) was unable to release its API content within the USP criteria of 30 min. The risk-based desirability function approach showed that 75% (3/4) of ciprofloxacin products were of good quality, and 25% (1) were found to be of acceptable quality, while the desirability function of norfloxacin tablets was found to be excellent, good, and acceptable; both accounted for 1 (33.3%). We wish to contend that, despite the apparent lack of difference between the outcomes of the conventional and D-function approaches, the D approach gives greater weight to the therapeutically more important quality traits and is hence appropriate for use in economically limited countries (51). Generally, this study revealed that the two selected expired products were of good quality in both the pharmacopeial and risk-based desirability function approaches to quality evaluation.

### Limitations and strength of the study

It’s understandable that with the limited scope of the study focusing on identity, assay, and dissolution parameters, there might be some constraints in drawing broader conclusions about the overall quality of expired pharmaceuticals in public health facilities. Including additional quality control parameters like weight variation, friability, and disintegration could indeed provide a more comprehensive assessment.

The study’s approach utilizing WHO sampling methods, USP standards, and FMEA methodologies reflects a robust methodology in pharmaceutical research. The findings regarding identity, assay, and dissolution criteria provide valuable insights into the quality of expired medications, challenging conventional assumptions about their efficacy post-expiration. This could have significant implications for healthcare policies, particularly in managing drug shortages and optimizing resource allocation.

## Conclusion, and recommendations

In the study, pharmacopeia, and failure mode, and effect analysis tools were attempted to evaluate the quality of expired Ciprofloxacin and Norfloxacin tablets for confirming medicines retained their quality after the shelf-life establishment date. Accordingly, all sampled items for quality evaluation contained the declared active ingredient and released their content as per USP pharmacopeial, but one ciprofloxacin batch was unable to release its API content within the USP criteria of 30 min. All sampled medicines contained their APIs within the pharmacopeial specification limit (100 ± 10). The risk-based analysis results indicated that assay was the most critical quality attribute. Additionally, the seven sampled expired medicines were within the risk-based desirability specification (0.70–1.00). Both pharmacopeial and risk-based approaches to quality evaluation of the products revealed that drugs had the potential to retain their quality after their expiration date.

Overall, the study’s findings highlight an important consideration regarding the expiration dates of pharmaceuticals. It’s evident that, under proper storage conditions, some drugs can retain their potency beyond their labeled expiration dates. This underscores the potential benefits of reassessing the quality and shelf-life of essential medicines, particularly in resource-constrained settings like Ethiopia where access and shortages are significant challenges. The direction for nationwide surveillance using pharmacopeial standards and FMEA-based approaches for expired medicines could be critical in ensuring continued access to quality medications. This approach not only supports healthcare sustainability but also aligns with global efforts to optimize pharmaceutical resource management.

## Data availability statement

The original contributions presented in this study are included in this article/Supplementary material, further inquiries can be directed to the corresponding authors.

## Author contributions

HG: Conceptualization, Data curation, Formal analysis, Investigation, Methodology, Validation, Writing – review & editing. SB: Conceptualization, Data curation, Formal analysis, Investigation, Methodology, Validation, Writing – review & editing. GH: Conceptualization, Data curation, Investigation, Methodology, Validation, Writing – review & editing. GT: Data curation, Resources, Visualization, Writing – review & editing. YTM: Data curation, Supervision, Validation, Writing – original draft, Writing – review & editing. SS: Project administration, Supervision, Visualization, Writing – review & editing.

## References

[1] Gikonyo D Gikonyo A Luvayo D Ponoth P. Drug expiry debate: The myth and the reality. *Afr Health Sci.* (2019) 19:2737–9. 10.4314/ahs.v19i3.49 32127846 PMC7040264

[2] Ashford N. Understanding technological responses of industrial firms to environmental problems: Implications for government policy. In: Fischer K Schot J editors. *Proceedings of the environmental strategies for industry: International perspectives on research needs and policy implications.* Washington DC: (1993).

[3] Kümmerer K. Pharmaceuticals in the environment. *Annu Rev Environ Resour.* (2010) 35:57–75. 10.1098/rstb.2013.0587 25405974 PMC4213597

[4] Adebisi Y Nwogu I Alaran A Badmos A Bamgboye A Rufai B Revisiting the issue of access to medicines in Africa: Challenges and recommendations. *Public Health Challenges.* (2022) 1:e9. 10.2147/CEOR.S413546 37332489 PMC10276598

[5] Guideline I. *Stability testing of new drug substances and products. Q1A (R2).* Amsterdam: European Medicines Agency (2003).

[6] Kamla P. Shelf life extension program: Revalidating expiry date. *J Appl Pharm.* (2016) 8:69–70.

[7] Ghaeli P. Drugs expiration date dilemma! *J Pharm Care.* (2014) 58:1–2. 10.31662/jmaj.2022-0209 38314420 PMC10834166

[8] Abramowicz M Zuccotti G Pflomm J. Drugs past their expiration date. *Med Letter Drugs Ther.* (2015) 57:164. 10.4314/ahs.v19i3.49 26633684

[9] Cantrell L Suchard J Wu A Gerona R. Stability of active ingredients in long-expired prescription medications. *Arch Intern Med.* (2012) 172:1685–7. 23045150 10.1001/archinternmed.2012.4501

[10] Lyon R Taylor J Porter D Prasanna H Hussain A. Stability profiles of drug products extended beyond labeled expiration dates. *J Pharm Sci.* (2006) 95:1549–60. 10.1002/jps.20636 16721796

[11] Diriba G Hasen G Tefera Y Suleman S. Assessment of the magnitude and contributing factors of expired medicines in the public pharmaceutical supply chains of Western Ethiopia. *BMC Health Serv Res.* (2023) 23:791. 10.1186/s12913-023-09776-y 37491251 PMC10367394

[12] Tura A Dalecha D Yasir M Ketebo T Noorulla K. Expiry of medicine in public health facilities of Arsi Zone, Oromia Regional State, Ethiopia: A quantitative and qualitative study. *Curr Issues Pharm Med Sci.* (2022) 35:27–33.

[13] Tull K. *Drug expiry standards in developing countries.* Taoyuan: KO DA Pharmaceutical Co Ltd (2018).

[14] Przyswa E. *Counterfeit medicines and criminal organisations.* Paris: IRCAM (2013).

[15] Ozawa S Chen H Lee Y Higgins C Yemeke T. Characterizing medicine quality by active pharmaceutical ingredient levels: A systematic review and meta-analysis across low-and middle-income countries. *Am J Trop Med Hyg.* (2022) 106:1778. 10.4269/ajtmh.21-1123 35895431 PMC9209904

[16] Tewuhibo D Asmamaw G Ayenew W. Availability of essential Medicines in Ethiopia: A systematic review. *J Commun Med Health Care.* (2021) 6:1049.

[17] Ebrahim A Teni F Yimenu D. Unused and expired medications: Are they a threat? A facility-based cross-sectional study. *J Prim Care Commun Health.* (2019) 10:2150132719847857. 10.1177/2150132719847857 31072253 PMC6537296

[18] Ziylan-Yavas A Santos D Flores EMM Ince NH. Pharmaceuticals and personal care products (PPCPs): Environmental and public health risks. *Environ Prog Sustain Energy.* (2022) 41:e13821.

[19] Basha S Babu K Madhu M Kumar Y Gopinath C. Recycling of drugs from expired drug products: Comprehensive review. *J Glob Trends Pharm Sci.* (2015) 6:2596–9. 10.1089/ars.2017.7234 29943652

[20] Scholtissek C Webster R. Long-term stability of the anti-influenza A compounds—amantadine and rimantadine. *Antiviral Res.* (1998) 38:213–5. 10.1016/s0166-3542(98)00015-1 9754890

[21] FDA. *FDA updates warnings for fluoroquinolone antibiotics.* Silver Spring, MD: FDA (2016).

[22] Machado-Duque M Mercado-Gómez K Bernal-Chica M Uribe-Vélez S Machado-Alba J. Prescription and indications for the use of fluoroquinolones in a group of outpatients in Colombia. *Biomédica.* (2020) 40:382–90. 10.7705/biomedica.5103 32673464 PMC7505503

[23] Metz-Gercek S Maieron A Strauß R Wieninger P Apfalter P Mittermayer H. Ten years of antibiotic consumption in ambulatory care: Trends in prescribing practice and antibiotic resistance in Austria. *BMC Infect Dis.* (2009) 9:1–7. 10.1186/1471-2334-9-61 19439064 PMC2686702

[24] Tirfe M Alemu A Alemu W Woldearegay M Asfaw G Gerba H A three years antimicrobials consumption in Ethiopia from 2017 to 2019: A cross-sectional study. *PLoS One.* (2023) 18:e0284038. 10.1371/journal.pone.0284038 37023072 PMC10079031

[25] Getahun H Belew S Hasen G Tefera Mekasha Y Suleman S. Assessment of the extent and monetary loss in the selected public hospitals in Jimma Zone, Ethiopia: Expired medicine perspectives. *Front Med.* (2024) 11:1283070. 10.3389/fmed.2024.1283070 38435389 PMC10906092

[26] Kebede O Tilahun G Feyissa D. Storage management and wastage of reproductive health medicines and associated challenges in west Wollega zone of Ethiopia: A mixed cross-sectional descriptive study. *BMC Health Serv Res.* (2021) 21:1–11. 10.1186/s12913-021-06291-w 33794887 PMC8017678

[27] WHO. *WHO access, watch, reserve, classification of antibiotics for evaluation and monitoring of use. WHO/HMP/HPS/EML/2021.04.* Geneva: WHO (2021).

[28] Belafsky P Mouadeb D Rees C Pryor J Postma G Allen J Validity and reliability of the eating assessment tool (EAT-10). *Ann Otol Rhinol Laryngol.* (2008) 117:919–24. 19140539 10.1177/000348940811701210

[29] Bodas M Yuval L Zadok R Hess Z Haran B Kaplan M Shelf-life extension program (SLEP) as a significant contributor to strategic national stockpile maintenance: The Israeli experience with ciprofloxacin. *Biosecur Bioterror Biodefense Strat Pract Sci.* (2012) 10:182–7. 10.1089/bsp.2011.0107 22578017

[30] Hajimolaali M Asl A. Quality risk assessment production of beta lactams by FMEA model and fuzzy theory method. *Gen Med Open Access.* (2016) 4:1–3. 10.25258/IJPQA.5.3.1

[31] Davido B Michelon H Mamona C de Truchis P Jaffal K Saleh-Mghir A. Efficacy of expired antibiotics: A real debate in the context of repeated drug shortages. *Antibiotics.* (2024) 13:466. 10.3390/antibiotics13050466 38786194 PMC11117793

[32] Ramachandran S. Drugs past their expiration date. *JAMA.* (2016) 315:510–1. 10.4314/ahs.v19i3.49 26836735

[33] Arioua A Shaw D. Use of expired drugs: Patients benefits versus industry interest. *JAMA.* (2024) 7:1–4. 10.31662/jmaj.2022-0209 38314420 PMC10834166

[34] Ethiopian Food and Drug Authority. *Pharmaceutical sector assessment in Ethiopia.* Addis Ababa: Ethiopian Food and Drug Authority (2016).

[35] Newton P Green M Fernández F. Impact of poor-quality medicines in the ‘developing’world. *Trends Pharmacol Sci.* (2010) 31:99–101. 20117849 10.1016/j.tips.2009.11.005 PMC2845817

[36] US Pharmacopeia National Formulary, USP31/NF26. *United States pharmacopeial convention.* Rockville, MD: US Pharmacopeia National Formulary (2008).

[37] US Pharmacopeia National Formulary, USP38/NF33. *United States pharmacopeial convention.* Rockville, MD: US Pharmacopeia National Formulary (2015).

[38] Bozdag E Asan U Soyer A Serdarasan S. Risk prioritization in Failure mode, and Effect Analysis using Interval Type-2 Fuzzy Sets. *Expert Syst Appl.* (2015) 42:4000–15. 10.1016/j.eswa.2015.01.015

[39] Zilker M Sörgel F Holzgrabe U. A systematic review of the stability of finished pharmaceutical products and drug substances beyond their labeled expiry dates. *J Pharm Biomed Anal.* (2019) 166:222–35. 10.1016/j.jpba.2019.01.016 30660807

[40] Bikbulatov E Stepanova I. Harrington’s desirability function for natural water quality assessment. *Russian J Gen Chem.* (2011) 81:2694–704.

[41] Breda S Jimenez-Kairuz A Manzo R Olivera M. Solubility behavior and biopharmaceutical classification of novel high-solubility ciprofloxacin and norfloxacin pharmaceutical derivatives. *Int J Pharm.* (2009) 371:106–13. 10.1016/j.ijpharm.2008.12.026 19150493

[42] The European Commission. *The European commission decision 2002/657/EC specifies a tolerance criterion for an analyte retention time relative to the standard substance retention time.* Brussels: The European Commission (2002).

[43] Ozawa S Shankar R Leopold C Orubu S. *Access to medicines through health systems in low-and middle-income countries.* Oxford: Oxford University Press (2019). p. iii1–3. 10.1093/heapol/czz119 PMC6901066 31816069

[44] Nwokike J. *Regulatory reliance and post-marketing surveillance systems for safe and accelerated introduction of new medical products in low-and middle-income countries.* Plymouth: University of Plymouth (2023).

[45] Diven D Bartenstein D Carroll D editors. *Extending shelf life just makes sense. Mayo clinic proceedings.* Amsterdam: Elsevier (2015). 10.1016/j.mayocp.2015.08.007 26433928

[46] Derbie A Mekonnen D Woldeamanuel Y Abebe T. Azithromycin resistant gonococci: A literature review. *Antimicrob Resist Infect Control.* (2020) 9:1–7. 10.1186/s13756-020-00805-7 32811545 PMC7436955

[47] Omo-Emmanuel U Chinedum O Obeagu E. Evaluation of laboratory logistics management information system in HIV/AIDS comprehensive health facilities in Bayelsa State, Nigeria. *Int J Curr Res Med Sci.* (2017) 3:21–38. 10.2147/JMDH.S286981 33469300 PMC7812048

[48] Freitag G. Guidelines on dissolution profile comparison. *Drug Inf J.* (2001) 35:865–74. 10.3390/pharmaceutics13101703 34683995 PMC8539859

[49] Zarmpi P Flanagan T Meehan E Mann J Østergaard J Fotaki N. Biopharmaceutical implications of excipient variability on drug dissolution from immediate release products. *Eur J Pharm Biopharm.* (2020) 154:195–209. 10.1016/j.ejpb.2020.07.014 32681966

[50] Huang G Nie M Mak K. Web-based failure mode and effect analysis (FMEA). *Comput Ind Eng.* (1999) 37:177–80. 10.1016/S0360-8352(99)00049-2

[51] Suleman S Zeleke G Deti H Mekonnen Z Duchateau L Levecke B Quality of medicines commonly used in the treatment of soil transmitted helminths and giardia in Ethiopia: A nationwide survey. *PLoS Negl Trop Dis.* (2014) 8:e3345. 10.1371/journal.pntd.0003345 25473966 PMC4256469

