# Supplementary material for: Quality evaluation of selected expired fluoroquinolones medicines obtained from the public hospitals in Jimma zone, Oromia regional state, Ethiopia
Source: Front Med (Lausanne). 2024 Aug 7;11:1420146. doi: 10.3389/fmed.2024.1420146 (PMC11335507; doi:10.3389/fmed.2024.1420146)

**S1fig. Linear desirability functions for assay**


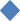

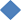

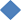

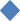

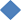

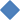

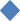


1.2

1

0.8

0.6

0.4

0.2

0

50 70 90 100 110 130 150

**% Label Claim**

**S2 fig. Linear desirability functions for dissolution**.


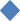

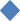

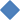

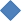

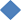

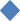

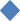


1.2

1

0.8

0.6

0.4

0.2

0

0

50

100

150

200

**% drug release**

**S3fig. Chromatogram of ciprofloxacin working standard(A), and samples(B)**.


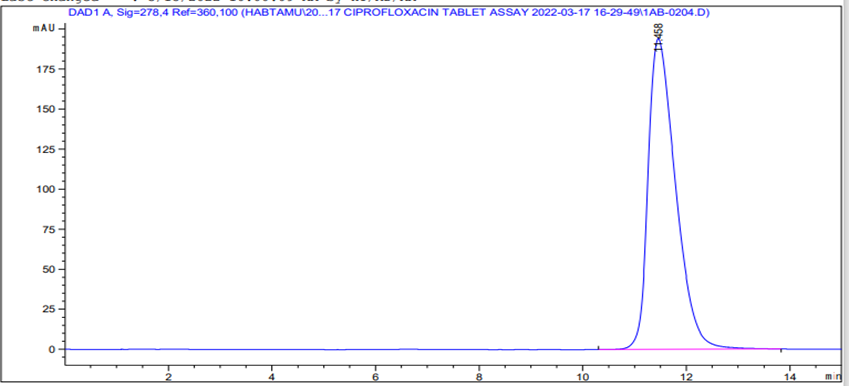
 A.


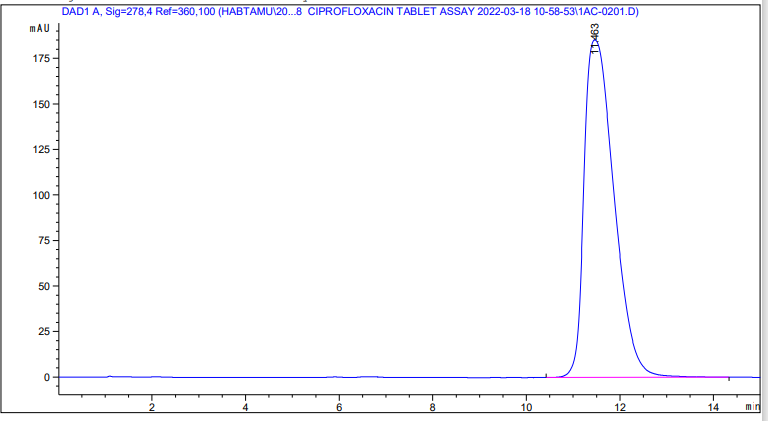
 B.

**S4fig. Calibration curve for ciprofloxacin working standard**.


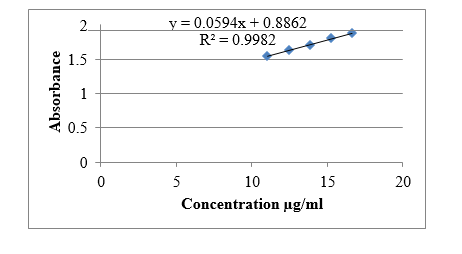

$$Y=mx+c$$

Where,

Y = absorbance reading of the UV-visible spectrophotometer, m = slope of the straight line, c = intercept on the AUC (y axis), and x = concentration of the analyte.

Hence, as depicted in figure 4, Y is absorbance, 0.0594 is the slope of the straight line, 0.8862 is the intercept on the area under the curve, and 0.9982 is linear regression.

**S5 fig. Chromatogram of norfloxacin working standard(A), and sample(B).**


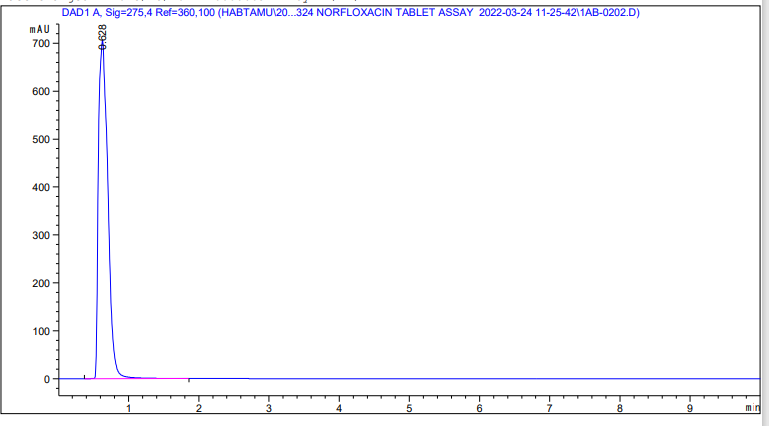
 A.


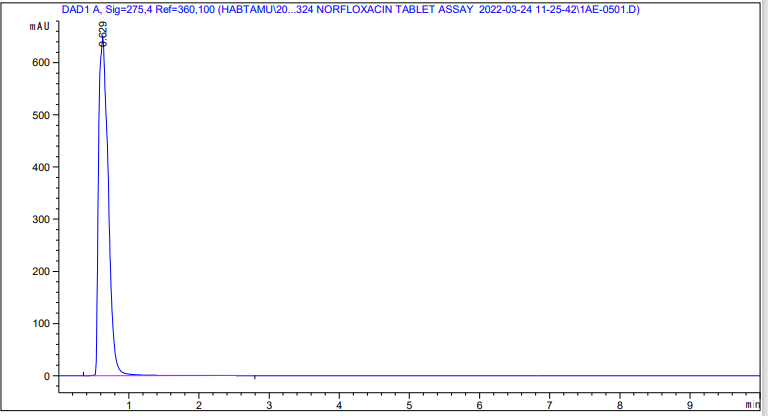
 B.

**S6 fig. Calibration curve for norfloxacin working standard**.


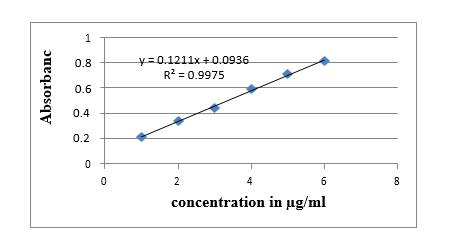

Supplement: Supplementary file 1 [file Data_Sheet_1.docx]
