# Supplementary material for: Quality evaluation of selected expired fluoroquinolones medicines obtained from the public hospitals in Jimma zone, Oromia regional state, Ethiopia
Source: Front Med (Lausanne). 2024 Aug 7;11:1420146. doi: 10.3389/fmed.2024.1420146 (PMC11335507; doi:10.3389/fmed.2024.1420146)
Supplement: Supplementary file 2 [file Data_Sheet_2.docx]

**S1 file. Sample collection protocol**

1. Area/Region/Country:
2. Sample code:
3. Name of location/place where sample was taken:
4. Names of people who collected the sample:
5. Product name of the sample:
6. Name of active pharmaceutical ingredient(s) (INN) with strength:
7. Dosage form (tablet, injection, powder for injection, etc.):
8. Package size, type and packaging material of the container:
9. Batch/lot number:
10. Date of manufacture: expiry date:
11. Name and address of the manufacturer:
12. Quantity collected (number of tablets and number of packages):
13. Comments on suitability of premises where products are stored, abnormalities, remarks or observation ns that may be considered relevant, if any:

Date:

Signature of person/s taking samples signature of representatives of the facility

where samples taken:

**S2 file. Details of sample information’s**

| Manufacturer | Brand name | Strength | Batch no | Manufacturing  date | Expiry date | Year after expiration,  year (months) |
| --- | --- | --- | --- | --- | --- | --- |
| Cadila pharmaceuticals Plc,  Ethiopia | Ciprodac | 500mg | D17024BY38 | 07/2017 | 06/2020 | 1 (8) |
| Cadila pharmaceuticals Plc,  Ethiopia | Ciprodac | 500mg | D14018BY38 | 4/2014 | 03/2017 | 4 (11) |
| BRAWN laboratories Ltd.,  India | Ciprofloxacin  (generic) | 500mg | BNT0418063 | 04/2018 | 03/2020 | 1 (11) |
| BRAWN laboratories Ltd.,  India | Ciprofloxacin  (generic) | 500mg | BNT0418048 | 04/2018 | 03/2020 | 1 (11) |

| Manufacturer | Brand name | Strength | Batch no | Manufacturing date | Expiry  date | Year after expiration,  year (months) |
| --- | --- | --- | --- | --- | --- | --- |
| Cadila Pharmaceuticals Plc,  Ethiopia | Norfen | 400mg | D15004BX64 | 04/2015 | 03/2018 | 3 (11) |
| East African Pharmaceuticals,  Plc, Ethiopia | Norflox | 400mg | IF108 | 06/2019 | 05/21 | 0 (9) |
| East African Pharmaceuticals,  Plc, Ethiopia | Norflox | 400mg | IF104 | 06/2019 | 05/21 | 0 (9) |

**S3 file. Individual desirability function of ciprofloxacin, and norfloxacin tablets**

| Identity | | | |  | Assay |  |  | Dissolution | |  |
| --- | --- | --- | --- | --- | --- | --- | --- | --- | --- | --- |
| S.No | Sample code | Identity | d-Identity | %LC | d-Assay (110- 100%lc) | d-Assay | %drug release at 30 minutes | d- dissolution (60-80%) | d- dissolution (80-100%) | d- dissolution |
| 1 | C-001 | complies | 1 | 103.35 | 0.90 | 0.90 | 94.29 |  | 0.91 | 0.91 |
| 2 | C-002 | complies | 1 | 104.69 | 0.86 | 0.86 | 78.67 | 0.67 |  | 0.67 |
| 3 | C-003 | complies | 1 | 101.23 | 0.96 | 0.96 | 87.25 |  | 0.81 | 0.81 |
| 4 | C-004 | complies | 1 | 100.87 | 0.97 | 0.97 | 83.88 |  | 0.76 | 0.76 |

| Identity | | | |  | Assay |  |  |  | Dissolution | |  |
| --- | --- | --- | --- | --- | --- | --- | --- | --- | --- | --- | --- |
| S.No | Sample code | Identity | d- Identity | %LC | d-Assay (90- 100%lc) | d-Assay (70-  90%lc) | d-assay | %drug release at 30 minutes | d- dissolution (80-100%) | d- dissolution (100-  110%) | d- dissolution |
| 1 | N-005 | complies | 1 | 90 |  | 0.70 | 0.70 | 101.84 |  | 0.83 | 0.83 |
| 2 | N-006 | complies | 1 | 94.12 | 0.82 |  | 0.82 | 94.65 | 0.92 |  | 0.92 |
| 3 | N-007 | complies | 1 | 99.55 | 0.99 |  | 0.99 | 93.84 | 0.91 |  | 0.91 |
